# Transcription as source of genetic heterogeneity in budding yeast

**DOI:** 10.1002/yea.3926

**Published:** 2024-01-09

**Authors:** Baptiste Piguet, Jonathan Houseley

**Affiliations:** ^1^ Epigenetics Programme Babraham Institute Babraham UK

**Keywords:** BIR, break‐induced replication, copy number variation, DNA repair, R‐loops, transcription‐replication conflicts

## Abstract

Transcription presents challenges to genome stability both directly, by altering genome topology and exposing single‐stranded DNA to chemical insults and nucleases, and indirectly by introducing obstacles to the DNA replication machinery. Such obstacles include the RNA polymerase holoenzyme itself, DNA‐bound regulatory factors, G‐quadruplexes and RNA‐DNA hybrid structures known as R‐loops. Here, we review the detrimental impacts of transcription on genome stability in budding yeast, as well as the mitigating effects of transcription‐coupled nucleotide excision repair and of systems that maintain DNA replication fork processivity and integrity. Interactions between DNA replication and transcription have particular potential to induce mutation and structural variation, but we conclude that such interactions must have only minor effects on DNA replication by the replisome with little if any direct mutagenic outcome. However, transcription can significantly impair the fidelity of replication fork rescue mechanisms, particularly Break Induced Replication, which is used to restart collapsed replication forks when other means fail. This leads to de novo mutations, structural variation and extrachromosomal circular DNA formation that contribute to genetic heterogeneity, but only under particular conditions and in particular genetic contexts, ensuring that the bulk of the genome remains extremely stable despite the seemingly frequent interactions between transcription and DNA replication.

## Take‐away

- Transcription can increase genetic heterogeneity or repair damage.
- Transcription can impact DNA replication fidelity in many ways but…
- …DNA replication forks very effectively traverse transcribed regions.
- Repair of forks by break‐induced replication can cause genetic heterogeneity.
- Transcription‐break‐induced replication interactions are highly mutagenic.

## Graphical abstract

Transcription can increase genetic heterogeneity or repair damage, and interactions with DNA replication are particularly challenging. Nonetheless, DNA replication forks effectively traverse transcribed regions, and we argue that replication–transcription conflicts are only mutagenic when DNA replication fork repair events interact with transcription.

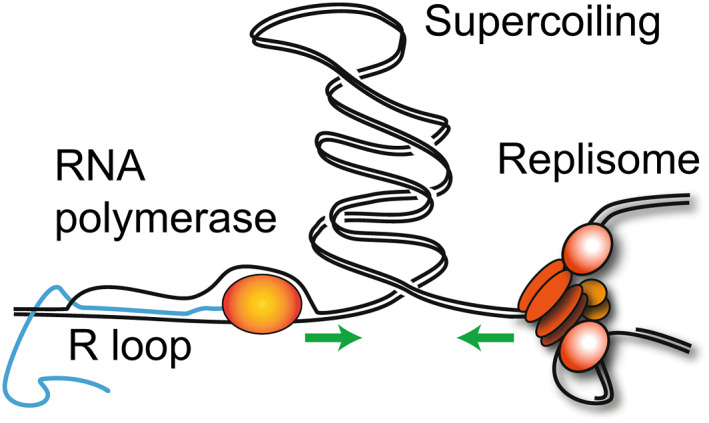

At first sight, the mode of action of RNA polymerase II appears detrimental to genome integrity. A simple model would predict that the transcribing holoenzyme follows the helical turns of the template strand, displacing histones and creating a single‐stranded bubble in a very localised region, but causing minimal disruption to surrounding genome topology. However, RNA polymerase II instead passages DNA through the active site, creating positive supercoils ahead of the holoenzyme and negative supercoils behind that must be either resolved by topoisomerases (Brill & Sternglanz, 1988) or allowed to relax when the polymerase dissociates (Tsao et al., 1989). The mechanism of this remains unclear: even bacterial RNA polymerase II working on a naked DNA can induce supercoiling (Janissen et al., 2023), but the outcome facilitates the release of the nascent RNA that would otherwise become wound around the DNA. Nonetheless, this behaviour also creates a set of vulnerabilities for mutation (Figure 1a):

1. Transcription requires the DNA helix to be transiently melted, and the single‐stranded DNA (ssDNA) formed is more sensitive to chemical insults and enzymatic modification than double‐stranded DNA (dsDNA).
2. Supercoiling around the polymerase must be relaxed by topoisomerases, which can be mutagenic when topoisomerase action is not completed.
3. The transcription regulatory machinery and RNA polymerases must be removed to allow passage of replication forks.
4. Negative supercoiling behind the polymerase enhances the formation of non‐B DNA structures, such as G‐quadruplexes and RNA‐DNA hybrids called R‐loops, which are considered to be a threat to genome stability.

**Figure 1:**
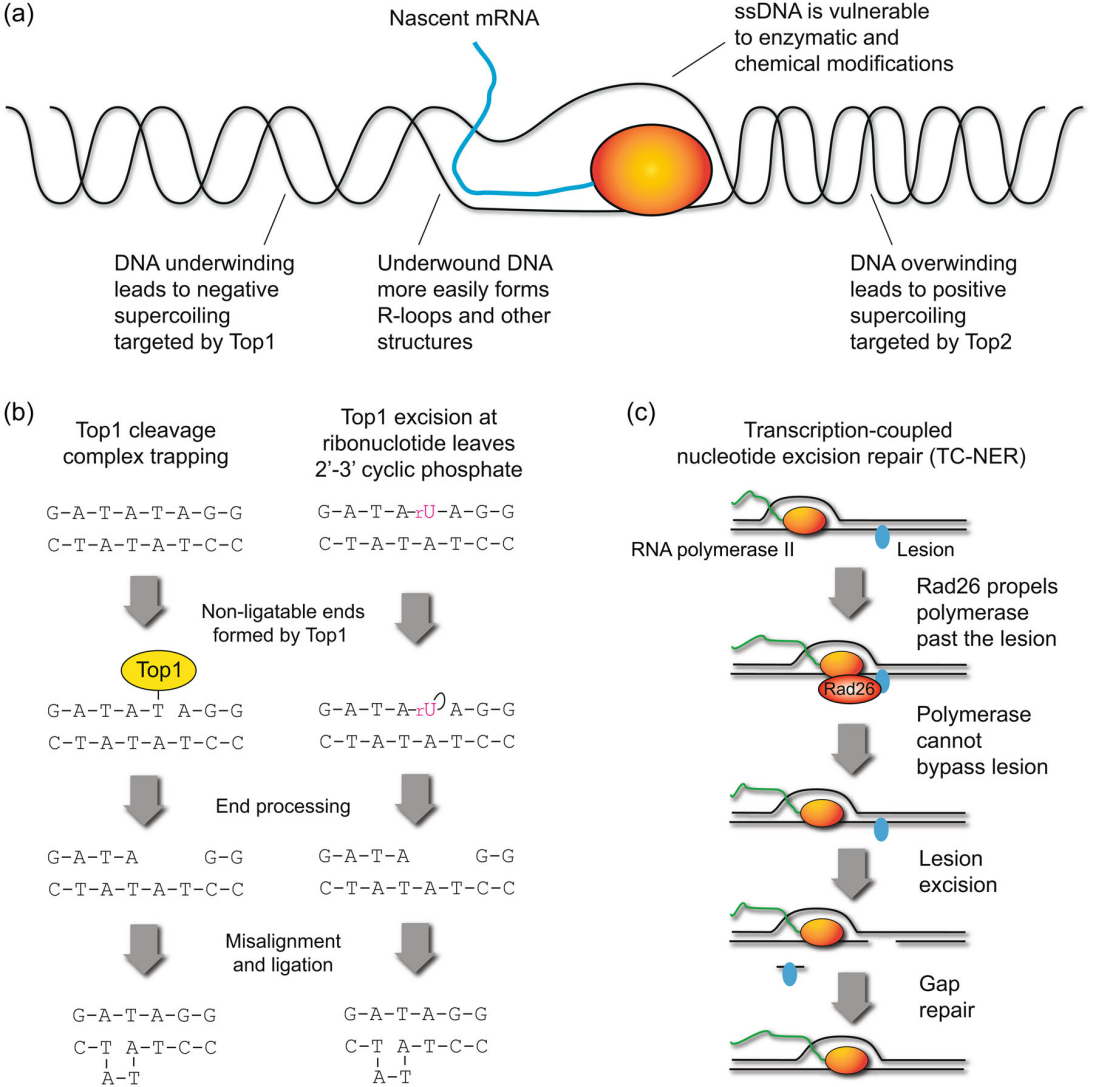
Vulnerabilities and opportunities for DNA integrity in transcription. (a) Transcription generates over‐wound DNA ahead of the RNA polymerase and under‐wound DNA behind the replication fork, forming positive and negative supercoils, respectively, that are substrates for different sets of topoisomerases. (b) Mutagenic potential of Top1 acting in short repeats: either accidental trapping of the Top1 cleavage complex or activity of Top1 at mis‐incorporated ribonucleotide forms non‐ligatable ends that must be excised. Misalignment of repeats allows ligation of mis‐matched ends, forming a DNA loop that is a substrate for mismatch repair or can become fixed by DNA replication. (c) Schematic of TC‐NER pathway acting on the template strand, showing outcomes for lesions that can be bypassed by RNA polymerase and for lesions that cannot.

Many of these vulnerabilities arise from interactions between DNA replication and direct or indirect outcomes of transcription, which must be resolved rapidly as the replisome needs to traverse many transcriptional units with minimal pausing to duplicate chromosomes without becoming a hindrance to cell growth and therefore fitness. Here, we review the mechanisms by which transcription can induce DNA damage in the budding yeast *S. cerevisiae*, focusing particularly on interactions with DNA replication.

## 1 TRANSCRIPTION PROVIDES MUTATIONAL VULNERABILITIES AND OPPORTUNITIES FOR REPAIR

Although transcription generates torsional stress, yeast RNA polymerases are less sensitive to this than we might expect: even cells lacking both topoisomerase I (Top1) and topoisomerase II (Top2) activity show defective RNA polymerase I transcription but surprisingly minor effects on RNA polymerase II and RNA polymerase III (Brill & Sternglanz, 1988; Brill et al., 1987). In‐depth studies revealed that only medium to long RNA polymerase II genes (>~3 kb) are affected, as well as the 6.7 kb RNA polymerase I transcript, as a result of accumulated positive supercoils ahead of the polymerase that is normally removed by Top2 (El Hage et al., 2010; Joshi et al., 2012).

Transcription‐induced positive supercoiling at convergent genes has not been associated with genetic changes, but negative supercoiling, which is resolved by Top1, does promote chromosome rearrangements (Pannunzio & Lieber, 2016; Trigueros & Roca, 2002). Furthermore, Top1 errors in the form of short deletions form up to 50% of mutations in highly transcribed sequences (Lippert et al., 2011; Takahashi et al., 2011). These arise both through the mis‐processing of trapped Top1 cleavage complexes in tandem repeats and through Top1 cleaving at ribonucleotides (Figure 1b) (Cho et al., 2013). Ribonucleotides are mistakenly incorporated in the genome during replication but usually removed by RNase H2 (Huang et al., 2015; Williams & Kunkel, 2014; Williams et al., 2013), however processing of ribonucleotides by Top1 results in a non‐ligatable end subject to potentially error‐prone repair (Huang et al., 2015; Kim et al., 2011). Top1‐mediated mutations are therefore a significant danger to the integrity of coding sequences and Top1 overexpression in yeast has been associated with mutagenesis and increased DNA damage, though surprisingly Top1 mutations are biased to the non‐template strand (Cho et al., 2015; Sloan et al., 2017). In contrast, wild‐type Top2 activity has not been associated with mutagenesis at transcribed loci in yeast though in vitro experiments have shown that Top2 can be trapped on DNA by abasic sites and ribonucleotides, and TOP2β generates promoter damage in human cells (Kingma et al., 1995; Pommier et al., 2016; Wang et al., 1999).

The passage of RNA polymerase II along the template strand creates a bubble of ssDNA that is also likely to increase mutation on the non‐template strand as ssDNA is innately more prone to endogenous chemical damage and enzymatic modification. For instance, spontaneous depurination and depyrimidination occur four times more frequently in ssDNA than in dsDNA (Billen, 1990), cytosine deamination to uracil forms over 100‐fold faster (Lindahl, 1993), and base alkylation occurs more frequently in ssDNA (Fu et al., 2012). Moreover, recent genome‐wide studies have confirmed that the non‐template strand is more frequently altered by alkylating agents and through spontaneous cytosine deamination (Mao et al., 2017; Williams et al., 2023).

In contrast, the genetic stability of the template strand is enhanced by transcription due to efficient machinery for repairing DNA lesions that are encountered by RNA polymerase II (Figure 1c). Many lesions can block the progression of RNA polymerase including intra‐ and interstrand crosslinks, DNA–protein crosslinks, cyclopurines and abasic sites (Brooks et al., 2000; Fielden et al., 2018; Jung & Lippard, 2007; Tornaletti et al., 2006). When elongating RNA pol II encounters such impediments, the remodelling factor Rad26 binds to the polymerase and the DNA sequence upstream. The ATPase activity of Rad26 allows a forward translocation of RNA polymerase II, facilitating the bypassing of non‐bulky lesions or benign obstacles (Duan et al., 2020; Xu et al., 2017), but when transcription‐blocking lesions are encountered the holoenzyme can engage the transcription‐coupled nucleotide excision repair (TC‐NER) system. In yeast, TC‐NER is activated by Rad26 or occasionally the non‐essential Rpb9 RNA pol II subunit (reviewed in (Li, 2015)), depending on expression level (Duan et al., 2020; Li & Smerdon, 2002, 2004). When Rad26 fails in translocating the RNA polymerase forward, the cell initiates a repair process involving the excision of nucleotides surrounding the lesion. Then, DNA polymerases, including Polδ and Polε, are recruited to fill in the gap and the newly synthesised DNA is ligated before the transcription restarts (reviewed in Gregersen & Svejstrup, 2018).

Although yeast lacking Rad26 and therefore deficient in TC‐NER show no growth defect under ideal conditions (van Gool et al., 1994), the long‐term importance of this system in maintaining transcriptional homeostasis and suppressing mutations is underlined by the phenotypes of human diseases caused by TC‐NER mutations, including UV‐sensitive syndrome, Xeroderma Pigmentosum and Cockayne syndrome, which is usually caused by mutations in the human orthologue of Rad26 (Lans et al., 2019). Conversely, it has been speculated that one reason why eukaryotes including yeast undergo genome‐wide pervasive transcription is to allow RNA polymerase to survey the genome for replisome blocking lesions for repair by TC‐NER before DNA replication (Ljungman, 2022), and direct evidence exists for this in bacteria as well as for telomeres in budding yeast (Guintini et al., 2022; Martinez et al., 2022). Furthermore, a yeast mutant deficient in global NER but proficient for TC‐NER shows increased UV resistance when spurious transcription initiation is licensed, demonstrating the capacity of pervasive transcription to improve genome stability (Selvam et al., 2022).

Transcription therefore has an innately strand‐biased effect on genome stability, increasing damage on the non‐template strand particularly during mutagen exposure, but resolving lesions on the template strand. Transcription also engages repair enzymes that introduce errors and strand breaks which may be mutagenic if encountered by the replisome.

## 2 THE POTENTIAL FOR TRANSCRIPTION TO IMPAIR DNA REPLICATION

Since transcription does not stop entirely during S‐phase, direct collisions between RNA polymerases and replisomes could occur. Higher Eukaryotes have evolved strategies to temporally separate transcription from replication and in yeast there is evidence that the replisome transiently represses transcription (Meryet‐Figuiere et al., 2014; Tsirkas et al., 2022). Even so, the transcriptional machinery includes many DNA‐bound protein complexes that might impair the movement of the replisomes even if active RNA polymerases are removed.

Whereas bacterial genes tend to be ordered co‐directionally with DNA replication (Kunst et al., 1997; McLean et al., 1998; Rocha, 2003), in *S. cerevisiae* almost half of the genes are transcribed opposite to the normal direction of DNA replication (referred to as “head‐on” genes), so encounters between the replisome and RNA polymerases must be frequent (García‐Muse & Aguilera, 2016; Goehring et al., 2023). Also, unlike bacteria, there is little evidence that head‐on orientated genes in yeast have higher mutation rates so the eukaryotic replisome must be adept at traversing transcriptional units (Kim et al., 2007; Lang & Murray, 2011; Sankar et al., 2016). In theory, DNA damage systems could alleviate transcription‐replication collisions in head‐on genes, but the DNA damage response is global and delays replication throughout the genome when forks stall, which must represent an emergency response rather than the default behaviour (reviewed in McClure et al., 2022). Similarly, while genome‐wide profiling of DNA replication has provided evidence that the replisome pauses at highly expressed loci, the delays required for the replisome to wait until transcription completes at each head‐on gene would be substantial (often >1 min given average gene size and RNA pol II holoenzyme speed [Muniz et al., 2021]), whereas minimal pausing was detected (Azvolinsky et al., 2009; Claussin et al., 2022; Kara et al., 2021). Replication profiles show that in *S. cerevisiae* many replisomes travel 50 kb through regions containing 30 or more genes in a normal S‐phase lasting ~30 min (Kara et al., 2021), which is at the limit of travel given the measured yeast fork speed of 1.6–1.9 kb/min (Hodgson et al., 2007; Sekedat et al., 2010), so pauses must be short and infrequent. Therefore, the eukaryotic replisome must efficiently displace RNA polymerases, just as the bacterial replisome has been directly observed to do by electron microscopy (French, 1992).

Although one might imagine that head‐on collisions between replication and transcription are inherently recombinogenic, extensive studies of the budding yeast ribosomal DNA show otherwise. The unidirectional replication fork barrier (RFB) formed by Fob1 binds the ribosomal DNA downstream of the massively transcribed 35S region and prevents the replisome from meeting oncoming RNA polymerase I head‐on (Kobayashi & Horiuchi, 1996). However, replication forks stalled at the RFB are highly recombinogenic (Kobayashi et al., 1998; Stewart et al., 1989), and rDNA recombination rate is dramatically reduced both in *fob1*Δ cells lacking the RFB and in *tof1*Δ cells in which replication forks do not stop at the RFB (Defossez et al., 1999; Johzuka & Horiuchi, 2002; Kobayashi et al., 1998; Mohanty et al., 2009). Encounters between the replisome and RNA polymerase I in *fob1*Δ do not cause a growth defect or create any genetic dependency on DNA repair proteins, and *fob1*Δ cells are long‐lived unlike homologous recombination mutants which are very short‐lived (Defossez et al., 1999; Park et al., 1999). We examined replisome progression directly in *fob1*Δ cells using our recently described TrAEL‐seq assay, and observed that replisomes progress efficiently head‐on through the RNA polymerase I transcription region to meet oncoming replisomes travelling codirectionally with RNA polymerase I (Kara et al., 2021), and in a further study showed that stalled replisomes do not accumulate in *tof1*Δ mutants at the site where the replisome meets oncoming RNA polymerase I, nor does this site acquire ɣH2A (Keszthelyi et al., 2023). This is also true for RNA polymerase II as reversion assays using a head‐on or codirectional *lys2* frameshift allele failed to demonstrate any significant difference in the overall rate of reversion between both orientations (Kim et al., 2007). There are exceptions however, since tRNA genes transcribed by RNA polymerase III do impede the replisome in head‐on encounters, as detected by both 2D‐gels and genome‐wide methods and these encounters can be recombinogenic (Azvolinsky et al., 2009; Claussin et al., 2022; de la Loza et al., 2009; Deshpande & Newlon, 1996; Kara et al., 2021; Tran et al., 2017).

Regulatory proteins bound to DNA could also form obstacles for the replisome. The substantial machinery bound at promoters includes transcription factors and transcriptional activators (catalogued in (Rossi et al., 2021)), but as noted above, genome‐wide studies have revealed only the mildest impacts of these on replisome progression (Azvolinsky et al., 2009; Kara et al., 2021). Helicases, including Rrm3 and Pif1, assist the replisome in removing proteins, easing the path through difficult features including tRNAs (Azvolinsky et al., 2006, 2009; Claussin et al., 2022; Ivessa et al., 2003; Tran et al., 2017), and it has recently been found that replisome pausing at tRNA is primarily due to bound TFIIIC complex rather than transcription per se (Yeung & Smith, 2020). However, the mild replisome pausing at highly expressed RNA polymerase II genes is independent of Rrm3 (Azvolinsky et al., 2009), suggesting that the core replisome is able to remove protein obstacles associated with RNA polymerase II activity.

RNA polymerase II and associated factors therefore have the potential to impede DNA replication, and there is some evidence for this in extremely highly transcribed genes, but during rapid growth under nutrient‐rich conditions, the replisome is very proficient in removing such obstacles, despite transcription being highest under such conditions. RNA polymerase III genes do have an effect on the replisome, but *S. cerevisiae* has dedicated helicases to ensure that replisome pausing at tRNA is minimal.

## 3 R‐LOOPS AS A THREAT TO GENOME STABILITY

**Figure 2:**
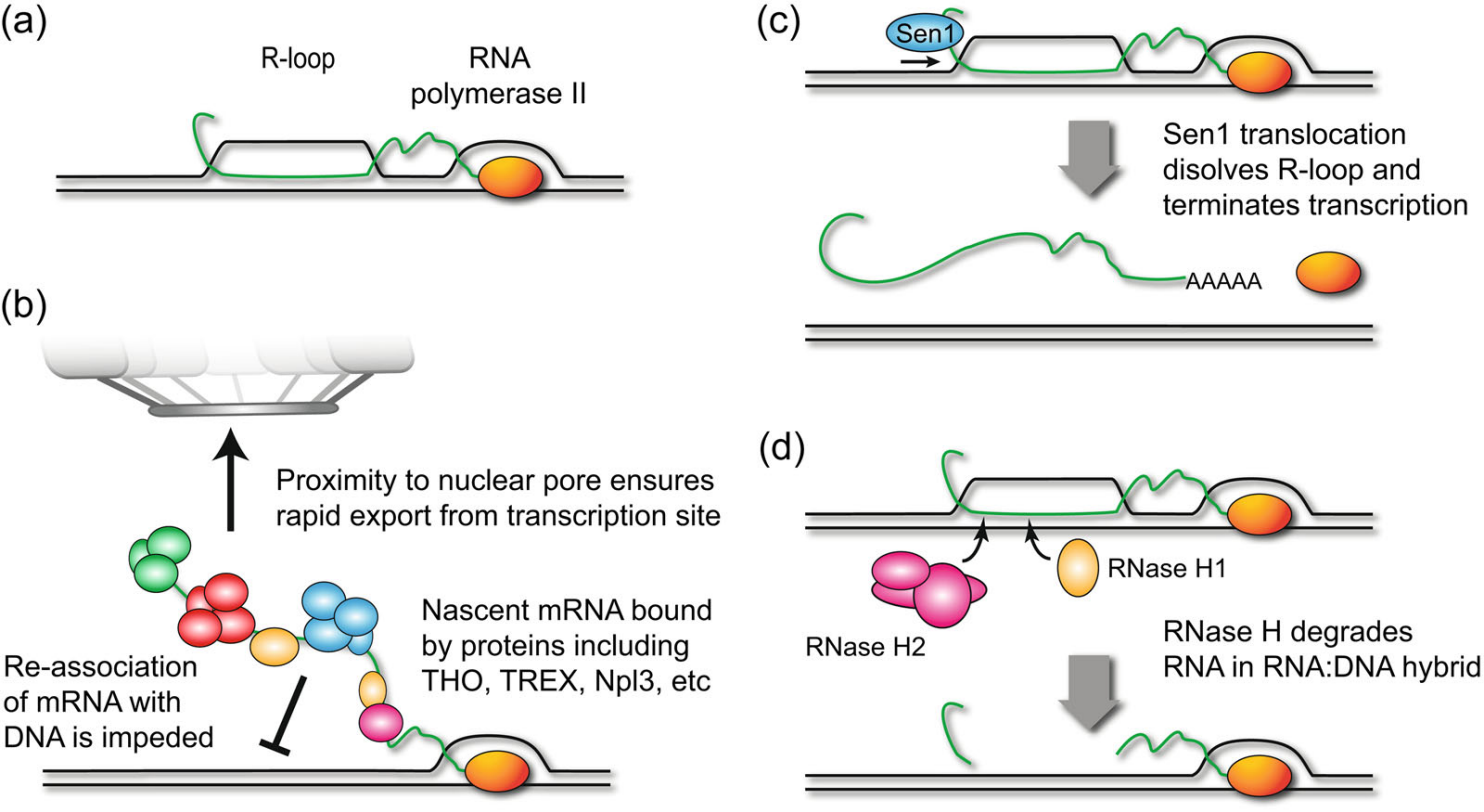
Defences against R‐loops. (a) Graphical representation of a co‐transcriptional R‐loop, formed by nascent DNA becoming bound to the template strand behind the polymerase. (b) Effect of mRNA binding and export factors in impairing R‐loop formation. (c) Activity of the termination factor Sen1 in removing R‐loops during translocation along the nascent mRNA toward the polymerase. (d) Activity of RNase H in degrading the RNA component of R‐loops, leaving fragments of mRNA to be degraded by exonucleases.

Transcription can result in the formation of R‐loops, in which the newly synthesised RNA hybridises with the DNA template strand before re‐annealing of the transcription bubble to form a stable DNA‐RNA hybrid structure (Figure 2a) (reviewed in Santos‐Pereira & Aguilera, 2015). R loops have physiological functions in transcription and in accurate chromosome segregation (Boque‐Sastre et al., 2015; Kabeche et al., 2018); however, the pathological accumulation of R‐loops has been repeatedly shown to increase genetic instability (reviewed in Brickner et al., 2022; Huertas & Aguilera, 2003; Neil et al., 2018). Genome‐wide profiling shows that R‐loops form more readily following RNA polymerase II pausing near transcription starting sites or transcription termination sites (TSS or TTS, respectively) but also form stochastically, particularly with RNA polymerase stalling or in regions of high GC content, and formation is facilitated by negative supercoiling accumulated behind RNA polymerase (Chan et al., 2014; El Hage et al., 2010; Skourti‐Stathaki et al., 2011, 2014; Wahba et al., 2016).

Budding yeast ameliorates the impact of R‐loops on genome stability by various mechanisms. First, coating of nascent mRNA with processing and export factors including THO/TREX reduces R‐loop formation and replication fork slowing (Garcia‐Rubio et al., 2018; Gomez‐Gonzalez et al., 2011; Luna et al., 2019; Wellinger et al., 2006), with THO/TREX mutants having unstable genomes (Prado, 1997; San Martin‐Alonso et al., 2021; Selvam et al., 2014). Another RNA‐binding protein, Npl3, which is involved in mRNA splicing and export in yeast, was also shown to decrease R‐loop dependent genomic instability and replication stress, and physical proximity of genes to nuclear pores which accelerates mRNA export was shown to reduce R‐loop accumulation (Figure 2b) (Gaillard et al., 2018; Garcia‐Benitez et al., 2017; Santos‐Pereira et al., 2013). Second, helicases can unwind R‐loops; the transcription termination factor Sen1 has a particular role in preventing R‐loop formation during S phase, with Sen1 depletion causing accumulation of both R‐loops and the ɣH2A histone marker which indicates the concomitant onset of DNA replication stress (Figure 2c) (Khurana & Oberdoerffer, 2015; Mischo et al., 2011; San Martin‐Alonso et al., 2021). Third, both RNase H1 and RNase H2 can degrade the RNA in R‐loops. Mutants lacking RNase H2 have a much greater impact on R‐loop mediated genome instability (O'Connell et al., 2015; Zimmer & Koshland, 2016), but RNase H1 has an ‘on‐demand’ activity at the critical time in S phase (Figure 2d) (Lockhart et al., 2019). Fourthly, chromatin modifiers including Rtt109 and FACT counter the formation of R‐loops (Canas et al., 2022; Herrera‐Moyano et al., 2014).

This multi‐layered defence against R‐loop formation and persistence is not surprising given the genome instability that results when cells with increased R‐loops undergo DNA replication. However, the mechanism by which R‐loops cause genome instability remains unclear as replicative helicases can unwind RNA‐DNA helices in vitro and replication forks can overcome DNA–RNA hybrids formed in co‐directional transcription units (García‐Rubio et al., 2018; Hamperl et al., 2017; Shin & Kelman, 2006). Moreover, hyper‐recombination phenotypes caused by R‐loops in yeast depend on Histone H3S10 phosphorylation suggesting an indirect mechanism involving chromatin (Garcia‐Pichardo et al., 2017). One possible explanation is that in head‐on encounters, the RNA polymerase becomes trapped between the R‐loop and the replisome, particularly in the absence of Sen1; this could impair normal mechanisms for removing the RNA polymerase holoenzyme from DNA in front of the replisome and form a potent roadblock (Felipe‐Abrio et al., 2015; Zardoni et al., 2021). Alternatively, as noted above, ssDNA is more prone to base damage, which would therefore accumulate on the non‐template strand in transcriptional R‐loops. However, experiments to address this mechanism discovered lesions arising from the replication bubble, but there was no dependence on R‐loops (Williams et al., 2023). Finally, R‐loops can be targeted by the TC‐NER or global genome NER pathway, resulting in cleavage and removal of the RNA‐DNA duplex followed by gap repair (Crossley et al., 2023; Sollier et al., 2014), which forms a short‐lived ssDNA gap that could be processed into a DSB by DNA replication (Vrtis et al., 2021).

Negative supercoiling behind RNA polymerase can also promote the formation of G‐quadruplexes—four‐stranded nucleic acid structures made between several guanosines, often stabilised by monovalent cations (Selvam et al., 2014). These structures can form throughout the *S. cerevisiae* genome and particularly at telomeres though they are not required for telomere function (Esnault et al., 2023; Skourti‐Stathaki et al., 2014). Like R‐loops, these structures have been implicated in replication fork stalling and leading to genome instability, and are removed by the helicase Pif1 (Lopes et al., 2011; Paeschke et al., 2011; Piazza et al., 2010).

Interruption of replisome progression by R‐loops and G‐quadruplexes can be recombinogenic, and may contribute to genetic heterogeneity even in unperturbed cells in a transcription‐dependent manner. However, R‐loops and G‐quadruplexes are common – the fact that these species can be detected in genome‐wide assays even in wild‐type yeast, indicates that at any given site, they are present in a significant fraction of cells. If each R‐loop even delayed the replisome, let alone caused a time‐consuming and mutagenic repair process, this would dramatically slow DNA replication irrespective of mutational burden. In reality, even RNase H1/H2 double mutants in which R loops are highly stabilised are viable and do not show a substantial growth defect (Zhao et al., 2018), suggesting that at most only a tiny fraction of R‐loops actually perturb replisome progression. Stalled replication forks can be rescued by converging forks from other origins, but the next origin can be very far away (up to a 95 kb region containing 55 genes in the *S. cerevisiae* genome), so the oncoming fork could also encounter an impediment necessitating rescue by firing of a dormant origin. A recent study shows that there are many more dormant origins than previously thought, coherent with the prediction that the distribution of origins in yeast genomes is optimised for robustness against replication impediments (Foss et al., 2023; Newman et al., 2013); however, we consider that frequent stalling of replication forks at such impediments even if robustly and effectively rescued would slow the completion of S phase and thereby impair competitive fitness.

## 4 MECHANISMS FOR ENSURING REPLISOME PROCESSIVITY

The replisome includes separate polymerase machineries for the leading and lagging strands, and the replicative helicase complex (Cdc45‐MCM‐GINS or CMG) which removes obstacles and unwinds DNA in front of the polymerases, as well as numerous auxiliary and regulatory factors (reviewed in Pellegrini, 2023). In unperturbed replication, the polymerase machineries are connected to the CMG to ensure that the replisome travels as a unit and to allow regulation of fork progression rate (Gambus et al., 2006; Jones et al., 2021; Simon et al., 2014).

**Figure 3:**
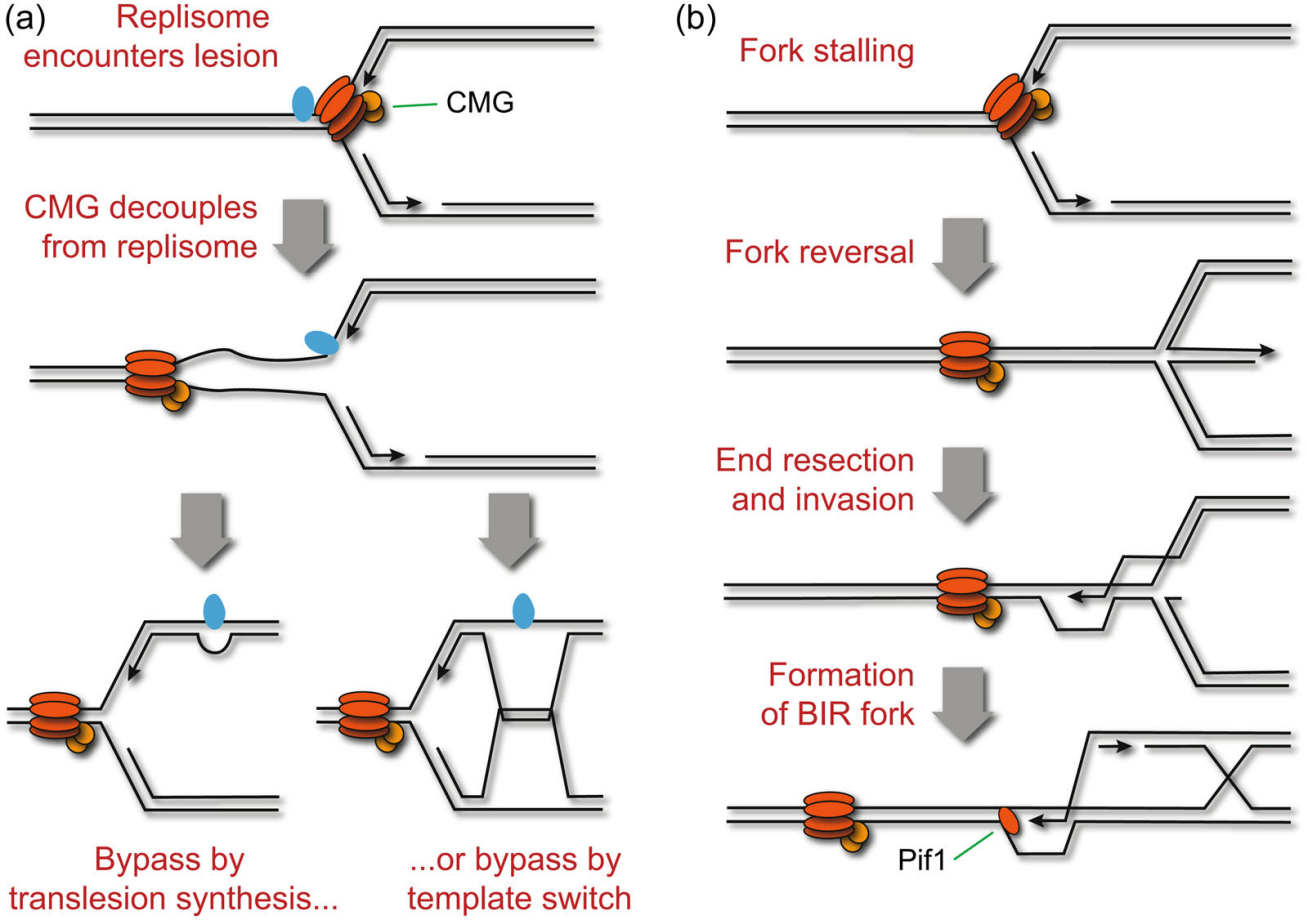
Replication fork impediments and processing pathways. (a) Replication past a polymerase‐blocking lesion: first, the leading strand polymerase stalls, but the replisome continues forming a region of ssDNA ahead of the fork. The polymerase can be restarted either by trans‐lesion synthesis or by template switching to use the newly synthesised lagging strand as a template. (b) Initiation of BIR: a stalled replication fork reverses, is resected then invades the template DNA aided by the homologous recombination machinery. Replication can then restart in a migrating D‐loop with uncoupled synthesis of the lagging strand, using Pif1 as a helicase to improve processivity.

When the replisome encounters an obstacle, either the CMG complex is uncoupled and continues along the template while one or both of the polymerases pause, or the whole replisome pauses. The former outcome results in the characteristic signature of replication stress (Figure 3a)—an accumulation of single‐stranded DNA between the DNA polymerases and the CMG complex. Persistent accumulation of ssDNA around the replication fork is recognised by the kinase Mec1 (the yeast homologue of ATR) via Ddc2 and the 9‐1‐1 complex, which then phosphorylates histone H2A at S129 in neighbouring chromatin, forming a focus of ɣH2A (Feng et al., 2006; Lopes et al., 2006; Namiki & Zou, 2006; Pardo et al., 2017; Puddu et al., 2011).

For base lesions such as 8‐oxo‐2′‐deoxyguanosine (8‐oxo‐dG), the DNA polymerases in the replisome are replaced with trans‐lesion synthesis enzymes Rad30 (Polη), Rev3/7 (Polζ) and/or Rev1, allowing DNA replication to continue past the lesion while (hopefully) inserting the correct base, with the lesion itself being left in place for NER or BER (Base Excision Repair) to cure post‐replication (reviewed in Powers & Washington, 2018; Zhao & Washington, 2017) (Figure 3a). Alternatively, template switching can be used to bypass lesions. In this process, the stalled polymerase temporarily switches to using the other newly synthesised DNA strand as a template to bypass obstacles (reviewed in Ripley et al., 2020) (Figure 3a). Both strategies aim to restart efficient DNA synthesis on both strands at the replication fork, resulting in a functional replication fork that is no longer associated with the replicative helicase, an equivalent situation to a fork paused for example by a short pulse of HU (Petermann et al., 2010). In both cases, replication appears to resume as normal suggesting that these replication forks re‐couple to the CMG helicase, probably facilitated by the slower progression of CMG that is uncoupled from the replicative polymerases (Graham et al., 2017; Petermann et al., 2010).

The CMG helicase can traverse almost all replisome obstacles, and can switch from a single to a double‐strand binding mode when uncoupled then back to single‐strand binding once re‐coupled (Wasserman et al., 2019). However, programmed replication fork barriers can block CMG progression resulting in a stable fork arrest, personified by the RFB in *S. cerevisiae* ribosomal DNA and *RTS1* at the *S. pombe* mating‐type locus (reviewed in Labib & Hodgson, 2007). This arrest is mediated not by a physical block to the replisome but by detection of the barrier by the Fork Protection Complex (FPC), which travels with the CMG complex (reviewed in Shyian & Shore, 2021). Similarly, the DNA replication checkpoint, which responds to global replication stress, can pause and stabilise replication forks without uncoupling them from the CMG complex, also through the FPC (Noguchi et al., 2004; Pardo et al., 2017), though at least in human cells and likely in yeast the forks become progressively less stable with time (Petermann et al., 2010).

Only if bypass and restart mechanisms fail, and additionally the fork is not resolved by an oncoming replication fork from another origin, must further potentially mutagenic options be explored. Once disconnected from the CMG helicase, a stalled fork gains the capability to reverse by re‐annealing the nascent leading and lagging strands to yield a 4‐pronged structure akin to a Holliday Junction and capable of migration over significant distances, a remodelling process assisted by Rad5 in budding yeast (Figure 3b) (Blastyak et al., 2007; Toth et al., 2022; Unk et al., 2010). Reversed replication forks gain a free double‐stranded end, which can be resected and initiate homologous recombination (Cotta‐Ramusino et al., 2005; Lemacon et al., 2017). Homologous recombination can restart the replication process, albeit in a different form known as Break‐Induced Replication (BIR) in which the leading strand is copied as a migrating D‐loop, then the lagging strand copied from the newly synthesised leading strand (Kramara et al., 2018; Liu & Malkova, 2022; Malkova & Ira, 2013) (Figure 3b). This marks a fundamental difference: whereas in canonical DNA replication both newly formed DNA helices contain one strand from the original DNA template (known as ‘semi‐conservative replication’), in BIR both strands of the daughter DNA helix are newly synthesised (‘conservative replication’). BIR uses a different DNA polymerase complement and relies on other helicases for processivity (notably Pif1) (Kramara et al., 2018; Liu & Malkova, 2022; Lydeard et al., 2010); movement of this complex is slow and error‐prone when forced to proceed over long distances, but in almost all instances this problem is avoided as the BIR fork is resolved by a replication fork coming from the other direction (Liu et al., 2021; Mayle et al., 2015). Because BIR is innately slow and error‐prone, replication forks are actively protected from resection so that transient fork pausing can be overcome with a non‐recombinational system that likely does not involve a loss of the replisome structure or function (Brambati et al., 2018). Single‐strand gaps are also converted to double‐strand breaks when encountered by the replisome and could enter a similar recombinational restart pathway (Vrtis et al., 2021). However, this must again be a last resort; mammalian cells use PARP to guard against this outcome and it is speculated that yeast use the FPC, at least for Top1‐mediated damage (Ray Chaudhuri et al., 2012; Westhorpe et al., 2020).

BIR forks have much more potential for introducing mutations than normal replication forks (Deem et al., 2011; Pardo & Aguilera, 2012; Sakofsky et al., 2014) as the lack of the CMG complex makes these prone to further stalling and recombinational repair if unresolved (Liu et al., 2021; Smith et al., 2007). Furthermore, proper sister chromatid cohesion requires replisome factors including the FPC, meaning that recombinational repair during BIR is much more likely to result in non‐allelic homologous recombination (van Schie & de Lange, 2021). Template switching based on microhomology is also more frequent, resulting in complex structural variations characterised by multiple switches with more or less homology at breakpoints (Pardo & Aguilera, 2012; Sakofsky et al., 2015). Given these properties, the slow progression of BIR forks is actively advantageous to the cell by providing the highest chance of resolution by a normal replication fork coming from the other direction and limiting the chances of mutation.

Therefore, stalled forks are protected from introducing further mutations at multiple levels. First, these forks are protected from processing by the recombination machinery for as long as possible; second, the recombination events are tightly restrained; and third, the low processivity of BIR forks favours resolution by oncoming forks.

## 5 KNOWN EXAMPLES OF TRANSCRIPTION‐INDUCED GENETIC CHANGE

Effective DNA repair of spontaneous damage and the excellent processivity of the replisome form a multi‐layered defence against potential mutations despite the considerable strain imposed by transcription. In keeping with this, mutation accumulation studies find only minimal evidence of a link between mutation and transcription (Chen & Zhang, 2014; Zhu et al., 2014), but clear examples of transcription‐induced mutation have been reported and mechanistic drivers have been elucidated.

**Figure 4:**
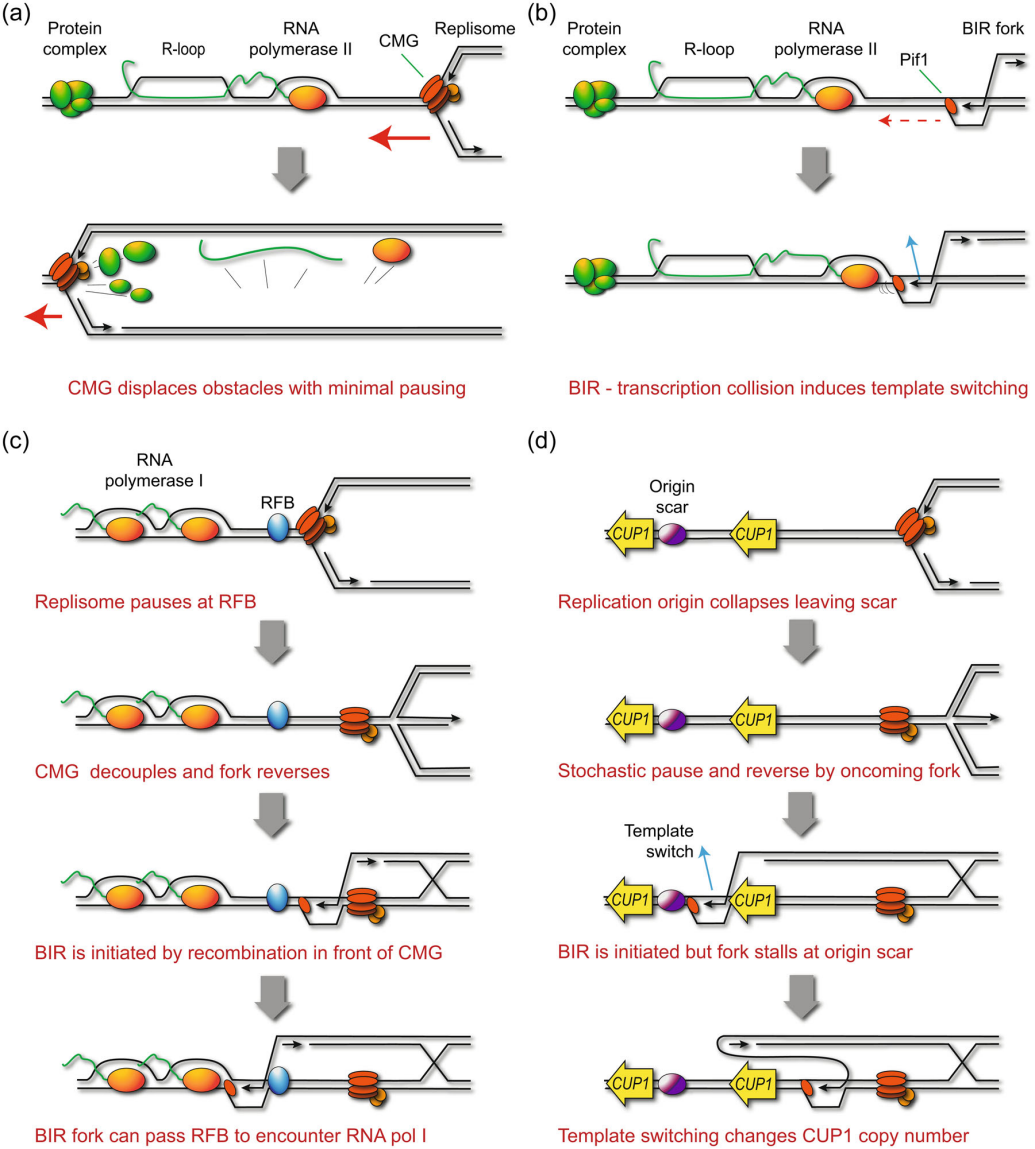
Mechanisms of genome instability arising through break‐induced replication (BIR). (a) In normal replication forks, CMG is the helicase that unwinds dsDNA. It has the ability to remove many types of impediments, including RNA‐DNA hybrids, RNA polymerases and other DNA‐bound proteins with minimal pausing, thereby allowing a fast progression of replication with short pausing times. (b) BIR forks are far more prone to template‐switching and to instigating non‐allelic recombination, likely because the helicase activity of Pif1 alone is far less than that of the CMG complex with assistance from Pif1 and Rrm3, making BIR more prone to template‐switch when encountering impediments. (c) Proposed model for transcription‐induced recombination at the RFB in the ribosomal DNA locus of budding yeast. Stabilised arrested forks can sometimes be restarted by BIR, and BIR forks are predicted to be insensitive to the RFB as RFB activity relies on the FPC, which is attached to the CMG complex that is lacking from BIR forks. BIR past the RFB puts the BIR fork in danger of head‐on encounters with elongating RNA polymerase I, leading to template‐switching, the formation of extrachromosomal ribosomal DNA circles and copy‐number variation of ribosomal DNA repeats. (d) Proposed model for CUP1 copy‐number variation. When encountering the H3K56ac scar left by a collapsed low‐efficiency replication origin inside the CUP1 repeats, a BIR fork could template‐switch to another CUP1 repeat and drive the formation of extrachromosomal CUP1 circles and copy‐number variation of CUP1 repeats. Note that the replisome stalling event which precipitates BIR does not need to be caused by transcription in this model, it is the collapse of the low‐efficiency origin that is caused by transcription.

To resolve this apparent contradiction, we propose a two‐step model: first, stalling of DNA replication forks either spontaneously or due to a replication–transcription conflict will occasionally require fork restart by BIR, but *de novo* mutation only arises through secondary events in which poorly processive BIR forks encounter obstacles resulting (for example) from transcription (illustrated in Figure 4a,b). Unlike replisome progression, direct measurements reveal BIR fork progression to be profoundly impaired by head‐on encounters with transcription, as well as with epigenetic marks to which the replisome is insensitive (Che et al., 2015; Liu et al., 2021). BIR forks are very prone to D‐loop dissociation and template switching when processivity is impaired, which would certainly explain transcription‐associated recombination events and may also underlie frameshifts (Deem et al., 2008; Smith et al., 2007, 2009; Vasan et al., 2014).

The best‐characterised example of transcription‐induced recombination in yeast occurs at the ribosomal DNA. It is currently thought that replication forks stalled at the ribosomal DNA RFB are cleaved, resected then undergo homologous recombination, causing copy number variation (CNV) if cohesin is removed by the transcription of non‐coding RNAs to facilitate recombination between repeats (Kobayashi & Ganley, 2005; Kobayashi et al., 2004). However, replication forks are stably paused at the RFB and it is unclear why these would be cleaved before resolution by oncoming replication forks (Carr & Lambert, 2013). Nonetheless, rDNA hyper‐recombination occurs in mutants lacking polymerase component Pol32 or deacetylase Hst3/Hst4, which should only impact the processivity of BIR forks, indicating that BIR is involved (Che et al., 2015; Houseley & Tollervey, 2011; Ide et al., 2013; Jack et al., 2015). Replication forks paused at the RFB are stable (Calzada et al., 2005), but the prominent one‐sided double‐strand break signal at the RFB indicates that replication fork reversal occurs, so resection and reinvasion of this end is likely to occur at some frequency (Burkhalter & Sogo, 2004; Kara et al., 2021; Zhu et al., 2019). The BIR events initiated are likely short‐lived due to resolution by oncoming forks, but could easily encounter oncoming RNA polymerase I and be forced into template switching. This would require the BIR fork to traverse the RFB, but given that the RFB is mediated by the FPC which is bound to CMG and that fork reversal dissociates the polymerases from CMG, this is plausible (Figure 4c).

Recently, we demonstrated that copper resistance acquisition in yeast arises through transcriptionally induced copy number amplification of the *CUP1* gene (Hull et al., 2017; Whale et al., 2022). The mechanism proved extremely complex, requiring a BIR event that becomes error‐prone on encountering an ‘epigenetic scar’ of H3K56ac chromatin which could arise from abortive replication initiation at a low‐efficiency origin upstream of the gene itself. Transcriptional dependence requires the activator complex Mediator and the mRNA export complex TREX‐2, which are recruited during activation of inducible genes such as *CUP1*. The only detectable TREX‐2‐dependent replication fork stalling at the locus occurred at the inefficient replication origin, suggesting that the primary impact of transcription is to prevent the successful firing of this origin. The unsuccessful origin firing leaves a scar of H3K56 acetylated chromatin, which causes BIR forks repairing local replisome stalling to undergo template switching, resulting in copy number amplification (Figure 4d). Importantly, this behaviour was not specific to the *CUP1* gene as the *SFA1* gene, which bestows formaldehyde resistance and like *CUP1* has an inefficient replication origin just upstream, can undergo an equivalent transcriptionally‐induced gene amplification through BIR (Hull et al., 2017).

It is worth noting that genetic changes induced by both these systems are not restricted to chromosomes. Both ribosomal DNA and *CUP1* loci are susceptible to transcription‐induced extrachromosomal circular DNA (eccDNA) formation. These species, particularly extrachromosomal ribosomal DNA circles (ERCs), accumulate to massive levels in aged cells, adding 30%–40% to genome size (Cruz et al., 2018; Hull et al., 2019; Sinclair & Guarente, 1997), and the ability of eccDNA to reintegrate in chromosomal DNA provides an additional pathway by which major transcription‐induced genetic changes can arise (Beverley et al., 1984; Brewer et al., 2015; Demeke et al., 2015; Galeote et al., 2011)

Interpretation of many studies in this area is complex due to the use of the extraordinarily highly expressed *GAL1* promoter; which is one of the few for which we can detect transcription‐induced pausing of the replisome by TrAEL‐seq (Kara et al., 2021). This may be sufficient to directly induce local BIR events at some frequency, and the pattern of transcription‐induced mutations induced in a *lys2* reporter is consistent with those reported to occur during BIR (Datta & Jinks‐Robertson, 1995). However, whether those mutations arise through conflicts between the BIR fork and transcription is hard to resolve if the rate of BIR induction is not necessarily constant, though follow‐on studies showed a direct correlation between transcriptional strength and reversion of *lys2* mutations, as well as induction of recombination events (Kim et al., 2007; Saxe et al., 2000), all consistent with BIR.

Overall, where it is possible to separate the direct impacts of transcription on the replisome and on BIR, the evidence is consistent with mechanisms in which transcription‐replisome conflicts are at most the initiator, whereas the actual mutations are caused by further conflicts impairing the processivity of BIR. We suggest that the danger posed by increased sensitivity of BIR forks to transcription‐related obstacles is the reason why transcription is globally downregulated in response to DNA replication stress (reviewed in Silva & Ideker, 2019). Notably, however, transcription‐induced genomic instability relies on specific properties of the ribosomal DNA, *CUP1* and *SFA1* loci, raising the question of whether some genes have evolved a configuration more prone to mutation or genome rearrangement.

## 6 OUTLOOK

Despite the potential for frequent interactions between transcription and DNA replication, wild‐type yeast cells grown under laboratory conditions have low enough rates of all mutation classes that populations grown from single cells are essentially clonal (Serero et al., 2014). This genome stability is useful but also problematic for yeast as colonising organisms: a few cells arriving in a compatible environment must rapidly reproduce, likely in competition with other yeasts and microorganisms, and then cells or spores from this population colonise new environments to complete the life cycle. The capacity for adaptive evolution is required to survive environmental differences and emerging threats from competing organisms as well as, where the colony forms in a living organism, host defences. However, populations arise from a few cells and have inherently low genetic heterogeneity, which limits evolutionary potential unless genetic heterogeneity is acquired during colony growth.

Our knowledge of interactions between DNA replication and transcription stems from studies under laboratory conditions, with unlimited nutrient availability and no competition. Under such conditions, the fidelity of DNA replication is extremely high, and the replisome is regulated to minimise replication fork stalling even under short‐term adverse conditions (Duch et al., 2013, 2018). However, long‐term exposure to environmental toxins such as ethanol does increase replication stress (Voordeckers et al., 2020), and we predict that genetic heterogeneity will then emerge in the population, particularly at environmentally responsive genes through increasing use and decreasing processivity of BIR forks. Yeast have therefore evolved to maintain a stable genome under favourable conditions but rapidly diversify the population under chronic stress by allowing interactions between transcription and DNA replication to become mutagenic.

## AUTHOR CONTRIBUTIONS

Baptiste and Houseley both conceived, wrote, edited and reviewed the manuscript. Data visualisation was done by Jonathan Houseley.

## Data Availability

Data sharing is not applicable to this article as no data sets were generated or analysed during the current study.
